# Studies of Grafted and Sulfonated Spiro Poly(isatin-ethersulfone) Membranes by Super Acid-Catalyzed Reaction

**DOI:** 10.3390/polym8040114

**Published:** 2016-03-29

**Authors:** Lei Jin, Hohyoun Jang, Jiho Yoo, Jaeseong Ha, Kunyoung Choi, Taewook Ryu, Sungkwun Lee, Whangi Kim

**Affiliations:** Department of Applied Chemistry, Konkuk University, Chungju 380-701, Korea; cici0204.lj@gmail.com (L.J.); figfight@hanmail.net (H.J.); jiho3340@naver.com (J.Y.); wotjd9608@naver.com (J.H.); Korea_355@naver.com (K.C.); gundam0924@naver.com (T.R.); skl@epchemtech.com (S.L.)

**Keywords:** PEM, super acid, spiro structure, dimensional stability, proton conductivity, carbon-carbon linkage

## Abstract

Spiro poly(isatin-ethersulfone) polymers were prepared from isatin and bis-2,6-dimethylphenoxyphenylsulfone by super acid catalyzed polyhydroxyalkylation reactions. We designed and synthesized bis-2,6-dimethylphenoxyphenylsulfone, which is structured at the meta position steric hindrance by two methyl groups, because this structure minimized crosslinking reaction during super acid catalyzed polymerization. In addition, sulfonic acid groups were structured in both side chains and main chains to form better polymer chain morphology and improve proton conductivity. The sulfonation reactions were performed in two steps which are: in 3-bromo-1-propanesulfonic acid potassium salt and in con. sulfuric acid. The membrane morphology was studied by tapping mode atomic force microscope (AFM). The phase difference between the hydrophobic polymer main chain and hydrophilic sulfonated units of the polymer was shown to be the reasonable result of the well phase separated structure. The correlations of proton conductivity, ion exchange capacity (IEC) and single cell performance were clearly described with the membrane morphology.

## 1. Introduction

Fuel cells are attractive renewable energy sources because they have efficiently high power densities, thereby making them ideal in the technology of the conversion of chemical into electrical energy. Nowadays, proton exchange membrane fuel cell (PEMFC) among five fuel cells is of great interest in vehicle application owing to the appropriate operation, high efficiency and wide applications [1,2,3]. Nafion^®^ and Flemion^®^ of a perfluorocarbon backbone are consisted, to which carbon-carbon main structure and fluorocarbon side chains are attached. These side chains carry a sulfonic acid group at their ends. It has been argued frequently in the literature that Nafion^®^ owes its good proton conductivity at relatively low water uptake to its morphology [4,5]. However, the disadvantages of the perfluorosulfonic acid polymers, such as high cost and low operation temperature (<100 °C) disrupt their applications [6,7,8]. Due to the several drawbacks of the the perfluorosulfonic acid polymers, the hydrocarbone membranes with low cost and high performance have been extensively studied [9,10,11,12,13,14,15,16,17,18]. 

At present, there are two key factors to improve proton conductivity and durability. The low proton conductivity of the membrane at low humidity appears to be the most critical factor and another important issue is membrane long term stability under severe condition of start-up and shut-down. Specially, hydrocarbon membrane showed very low proton conductivity at low RH compared to Nafion^®^.

To improve the conductivity under low humidity, block copolymers were suggested within which the sulfonic acids are locally oriented, and the formation of the hydrophobic domains prevents water solubility. Therefore, the microphase separations of block copolymers were formed by hydrophilic domain of highly sulfonated chain segments and hydrophobic domains formed by the nonsulfonated segments. Block copolymer membranes showed higher proton conductivity and lower water uptake than random copolymer membranes at the similar concentration of sulfonic acid [19,20,21,22,23,24]. To improve their durability, polyphenylene or carbon–carbon structures were suggested for which polymers without ether linkage in polymer main chain are derived from Diels–Alder, super acid catalyze and Ni/Zn catalyze reactions [25,26,27,28,29,30,31]. Diels–Alder polymerization has a limitation of high molecular weight and desirable monomer synthesis. Ni/Zn catalyze and super acid catalyze reactions deserve the most attention in future membrane development. Our research group has studied polymer structures without ether linkage using super acid [32,33,34]. The purpose of this work is to design and synthesize bis-2,6-dimethylphenoxyphenylsulfone, which is structured at the meta position steric hindrance by two methyl groups, because this structure minimized crosslinking reaction during super acid catalyzed polymerization, and also grafted sulfonic acid for improving proton mobility, low water uptake and good solubility in polar solvent. Clearly, attaching the acidic groups to the backbone via side chains can be expected to be beneficial [5,35].

This study aims to synthesize spiro polymer from isatin and bis-2,6-dimethylphenoxyphenylsulfone with trifluoromethane sulfonic acid as a catalyst, and the sulfonated polymer was followed by sulfonation concentrated sulfuric acid to prepare sulfonated polymer or grafting reaction with 3-bromo-1-propane sulfonic acid potassium salt which was prepared from propanesultone. The morphology of sulfonated membrane was depicted by AFM and high proton conductivity. The synthesized copolymers were characterized by ^1^H NMR spectroscopy, thermogravimetric analysis (TGA), proton conductivities, water uptake, and ion exchange capacity (IEC) measurement.

## 2. Experimental

### 2.1. Materials

Isatin (1H-indole-2,3-dione), 4,4′-Dichlorodiphenyl sulfone, 2,6-dimethylphenol, 1,3-propane sultone and potassium bromide were purchased from Aldrich. Trifluoromethansulfonic acid (TFSA) was purchased from Alfa Aesar (Lancashire, UK) and potassium carbonate and sodium sulfate from Acros Organics (Antwerpen, Belgium). *N*,*N*-dimethylacetamide (DMAc) was dried over calcium hydride for 24 h and distilled before use. Common solvents, such as chloroform, dichloromethane and hexane were also used without further purification.

### 2.2. Synthesis of 4,4-(2,6-dimethylphenyloxy)Diphenyl Sulfone (DMPPS)

**Scheme 1:**
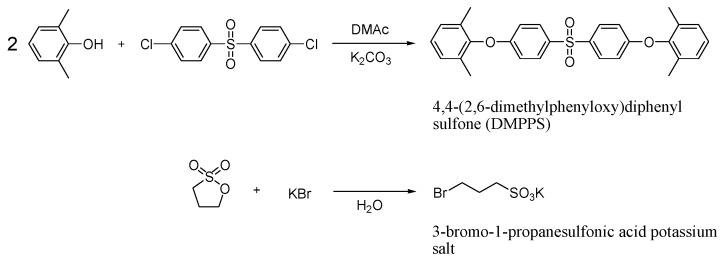
Preparation of monomers.

The synthesis of monomer was shown in Scheme 1. The monomer was conducted in a 100 mL one-neck flask equipped with a magnetic stirrer and condenser under N_2_ gas. In the monomer, 4,4′-dichlorodiphenyl sulfone (CPS) 10 g (34.82 mmol), 2,6-dimethylphenol 9.358 g (76.604 mmol) and potassium carbonate 10.587 g (76.604 mmol) were added into the flask. Subsequently, DMAc (60 mL) was added into the flask. The reaction mixture was heated up to 180 °C, and maintained for about 9 h under N_2_ atmosphere. The reaction mixture was diluted with dichloromethane and we used a glass filter to remove potassium carbonate. Then the products were washed with saturated NaCl water three times to remove DMAc. The organic layer was dried with MgSO_4_ and decolorized with activated charcoal. The filtrate was evaporated, and the obtained solid product was purified by recrystallization form chloroform/*n*-hexane to afford 4,4-(2,6-dimethylphenyloxy)diphenyl sulfone (DMPPS) in 84% yield. The monomer was a pale yellow powdered solid.

### 2.3. Preparation of Poly(isatin-ethersulfone)

**Scheme 2:**
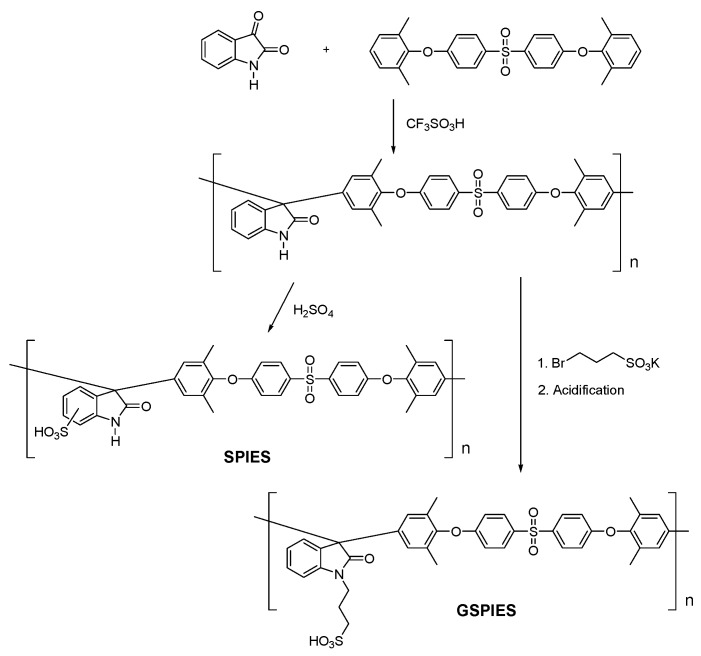
Preparation of sulfonated poly(isatin-ethersulfone) (SPIES) and grafting sulfonated poly(isatin-ethersulfone) (GSPIES).

The polymer was synthesized as shown in Scheme 2. The monomers were polymerized in a 250 mL two-neck flask equipped with a stirrer and dropping funnel. In a polymerization, isatin (3.25 g, 22.12 mmol) and DMPPS (7.80 g, 17.02 mmol) were added into the flask, and then dichloromethane (16.5 mL) and trifluoromethansulfonic acid (11.74 mL) were added into the mixtures. The reaction mixture was stirred at room temperature for 6 h until the solution became highly viscous. The solution was decanted into 100 mL of methanol to give a white fiber. The precipitation was repeatedly washed with methanol until the neutral pH and the polymer was dried at 60 °C for 24 h under vacuum.

### 2.4. Synthesis of Sulfonated Poly(isatin-ethersulfone) (SPIES)

The sulfonation was synthesized as shown in Scheme 2. Polymer (0.5 g, 0.850 mmol) was sulfonated with sulfuric acid (10 mL) as reagent and solvent in a 50 mL flask, and the mixture was reacted at 40 °C for 6 h. After completion of the reaction, the reaction mixture was poured slowly into 100 mL of cold deionized water, and the yellow fibrous product was filtered. The sulfonated polymer was washed with deionized water several times until the pH was neutral. The polymer was dried at 60 °C for 24 h in a vacuum oven.

### 2.5. Synthesis of 3-bromo-1-propanesulfonic Acid Potassium Salt

3-Bromo-1-propanesulfonic acid potassium salt was synthesized as shown in Scheme 1. A solution of 1,3-propane sultone (5 g, 40.94 mmol) and potassium bromide (5.85 g, 49.12 mmol) in distilled water (75 mL) was reacted at 60 °C for 1 h. The reaction mixture was evaporated to remove the water. The residue was washed with cool ethanol. The resulting product was recrystallized from ethanol and water (3:1 *v*/*v*), a white solid (8.88 g, 90%) was obtained.

### 2.6. Synthesis of Grafting Sulfonated Poly(isatin-ethersulfone) (GSPIES)

The grafting sulfonation was synthesized as shown in Scheme 2. The polymer was conducted with polymer (1.0 g, 1.701 mmol) and DMAc (15 mL) in a 100 mL one-neck flask with a sitter at 60 °C for 3 h. After polymer completely dissolved in DMAc, 3-bromo-1-propanesulfonic acid, potassium salt (0.62 g, 2.55 mmol) was added, and then added K_2_CO_3_ (0.35 g, 2.55 mmol). Then the mixture was stirred at 60 °C for 48 h. The solution was poured slowly into 100 mL of deionized water. The fibrous polymer was washed with deionized water several times until the residual water changed to neutral pH, and dried for 24 h at 60 °C. After the polymer was fully dried, we obtained 0.5 g of product, which we dissolved in DMAc to make a film and dried for 24 h at 80 °C in an oven. Then the film was absorbed in 1.0 M hydrochloric acid solution for 24 h at 80 °C. After complete acidification, the sulfonated film was washed with deionized water until the pH was neutral.

### 2.7. Membrane Preparation and Characterization

Membranes (25 µm) were prepared by the 20 wt % filtered polymer in DMSO to afford transparent film through the ambient temperatures of 60, 80, 100, 120 °C. The ^1^H NMR spectra were recorded on a Bruker DRX (400 MHz, Bruker, Billerica, MA, USA) spectrometer using tetramethylsilane (TMS) as an internal standard. The structure was confirmed by Fourier transform infrared (FT-IR) spectroscopy using a NICOLET FT-IR spectrometer (Thermo scientific Inc., Waltham, MA, USA). Thermogravimetric analyses were used Scinco TGA-N 1000 (Scinco, Chicago, IL, USA) and analyzed under the condition of a heating rate of 10 °C/min, from 30 to 800 °C. The dry and wet measurements of membranes were vacuum-dried at 100 °C for 24 h, weighed and immersed in deionized water at 30 °C and 80 °C for 24 h. The water uptakes of membranes were measured by three samples, and calculated as the ratio of adsorbed water on the dry sample as follows:

Water uptake (%) = [(*W*_wet_ – *W*_dry_)/*W*_dry_] × 100 (1)

where *W*_wet_ and *W*_dry_ are the weights of the wet and dry membranes, respectively. The ion exchange capacity (IEC) of the membrane acid function was obtained by the titration method as follows: The acid form (H^+^) of membrane was immersed in 20 mL of a 2.0 M NaCl solution at 80 °C for 24 h, and then converted to the sodium salt form. The proton conductivity is used a through-plane membrane test system (Scribner Associates Inc., Southern Pines, NC, USA, MTS 740) with a Newton’s 4th Ltd (N4L) impedance analysis interface (PSM 1735). The membrane was hydrated for 24 h at room temperature in deionized water before use, and then placed in a sealed cell. The proton conductivity was obtained by measuring the AC potential difference occurring at the middle of the membrane between two electrodes. The conditions were from 30 to 80 °C under 80% RH, and at 80 °C under 30%–90% RH while constant alternating current was applied to the both electrodes. Frequency dependence of the cell impedance was measured at 10 mV over the frequency of the alternating voltage from 1 to 1 × 105 Hz. Atomic force microscope (AFM) images were observed by PSIA XE100 with a Digital Instrument (Milano, Italy), SII-NT SPA400, using microfabricated cantilevers with a force constant of −20 N·m^−1^. The surfaces of the films of synthesized polymers was applied in non-contact mode with P/N 910M-NCHR tips. The measuring samples were maintained under 100% RH at room temperature for 24 h before use. Membrane electrode assembly (MEA) is composed of polymer electrolyte membrane (PEM), catalyst layers (CL) and gas diffusion layers (GDL) attached on the outer surface of the catalyst layers. The active area of 9 cm^2^ were prepared using a decal method with a 20 wt % wet-proofed Toray carbon paper (TGPH-060, Toray Inc., Tokyo, Japan) of 190 μm in thickness as a gas diffusion layer (GDL). Carbon supported 50% Pt (Hispec 13,100, Johnson Matthey Inc., London, UK) was 0.2 mg·Pt/cm^2^. Cell performance was performed by PEMFC test station (CNL Energy Co., Seoul, Korea). The molecular weight of the polymers was determined relative to polystyrene standards by gel permeation chromatography (GPC) in THF as the eluent on a PerkinElmer series 200 high pressure-liquid chromatographer (PerkinElmer, Billerica, MA, USA) and a RI-detector. The tensile stress-strain properties of the membranes were tested using a Com-Ten Industries 95 T series (Comten Industries Inc., Pinellas Park, FL, USA) load frame equipped with a 200 lbf load cell and computerized data acquisition software. Samples of 9 mm width were deformed at a crosshead speed of 5 mm/min with gauge length of 30 mm.

## 3. Results and Discussion

### 3.1. Charaterization of Monomer and Polymers

**Figure 1:**
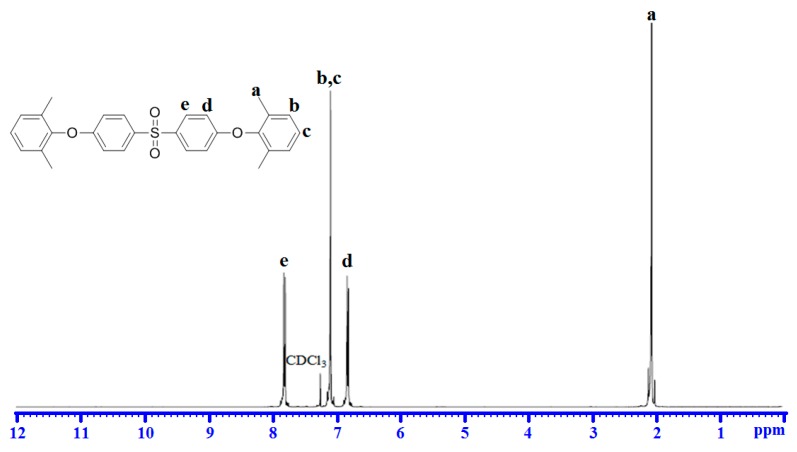
^1^H NMR of 4,4-(2,6-dimethylphenyloxy)diphenyl sulfone.

New reactive monomer, 4,4-(2,6-Dimethylphenyloxy)diphenyl sulfone (DMPPS), was synthesized and shown in Scheme 1. The substitution reaction of 2,6-dimethylphenoxy potassium salt and 4,4′-dichlorodiphenyl sulfone proceed step-wise because of steric hindrance of dimethyl groups. The protons of the synthetic monomer were identified by ^1^H NMR and described in Figure 1. The proton peaks of phenoxy-CH_3_ were characterized and appeared at 2.09 ppm, and six protons of phenyl rings appeared multiple peaks at 7.11 and 7.12 ppm, respectively. The proton peaks of diphenyl sulfone appeared at 7.83 and 6.86 ppm. Poly(isatin-ethersulfone) was successfully synthesized from isatin and DMPPS with trifluoromethansulfonic acid as a super acid. During the reaction, the crosslinking reaction did not occur because the dimethyl groups on ortho-positions protected the side reaction until high molecular weight was achieved. Sulfonation reactions were shown in Scheme 2. SPIES was obtained by concentrated sulfuric acid, and also GSPIES was prepared from the reaction of PIES and 3-bromo-1-propanesulfonic acid potassium salt, and then acidified with 1.0 M hydrochloric acid solution. The molecular weight of the polymers was determined by gel permeation chromatography (GPC). PIES showed a molecular weight (*M*_w_) range from 84,500 to 97,200, SPIES showed from 86,700 to 99,400 and GSPIES showed from 88,100 to 100,800.

**Figure 2:**
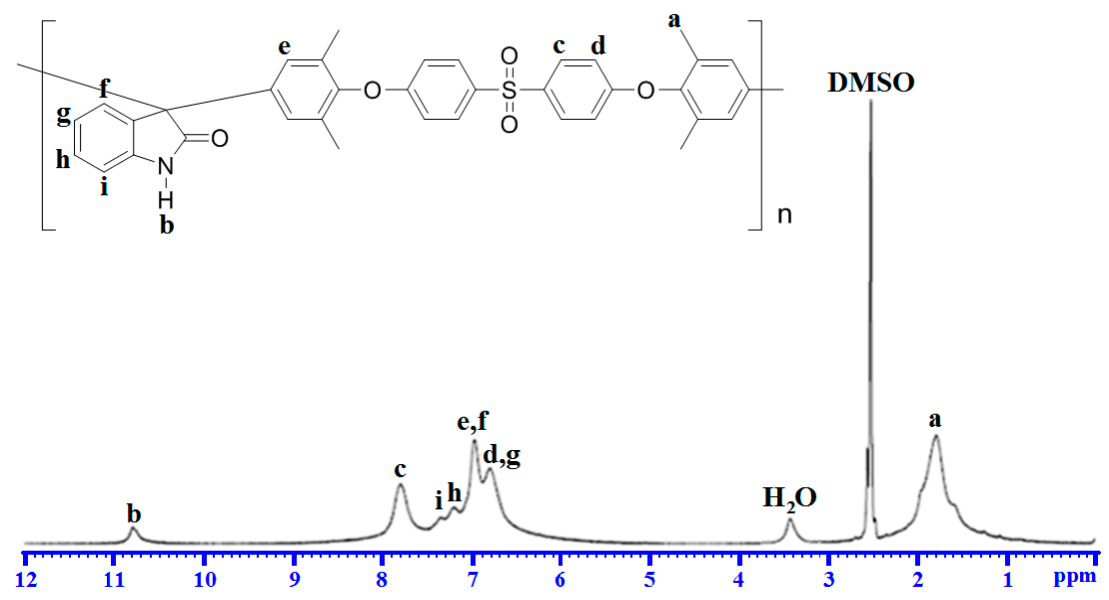
^1^H NMR of poly(isatin-ethersulfone) (PIES).

**Figure 3:**
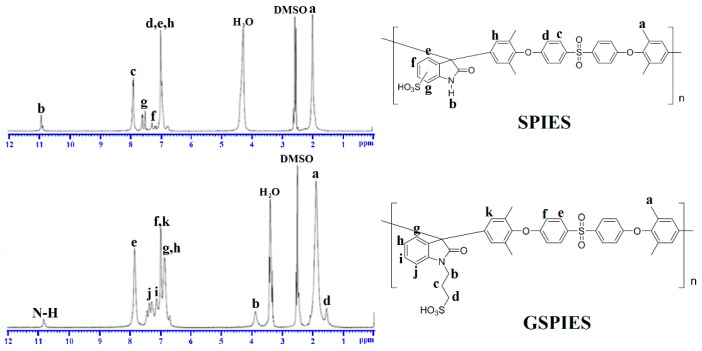
^1^H NMR of sulfonated poly(isatin-ethersulfone) (SPIES) and grafting sulfonated poly(isatin-ethersulfone) (GSPIES).

The verification of the polymer structure was identified by ^1^H NMR as shown in Figure 2 and Figure 3. the proton peaks of N–H assigned at 10.79 ppm. The alkyl groups of propanesulfonic acid appeared at 3.87, 2.46 and 1.52 ppm, respectively. Before reaction of 3-bromo-1-propyl sulfonic acid, the integration ratios of NH and phenyl H were 1 to 1.4. After reaction of 3-bromo-1-propyl sulfonic acid, the reaction was about 50% complete. The integration ratios of NH and phenyl H were 1 to 2.8. The protons of propyl agreed with the decrease of the NH peak. After sulfonation, the integration value of phenyl protons was reduced and the protons near the sulfonic acid group were shown at downfield due to the electron withdrawing effect of sulfonic acid. The peak at 4.3 ppm was shifted from 3.3 ppm because DMSO was formed on the conjugated acid as (DMSO)-H^+^.

**Figure 4:**
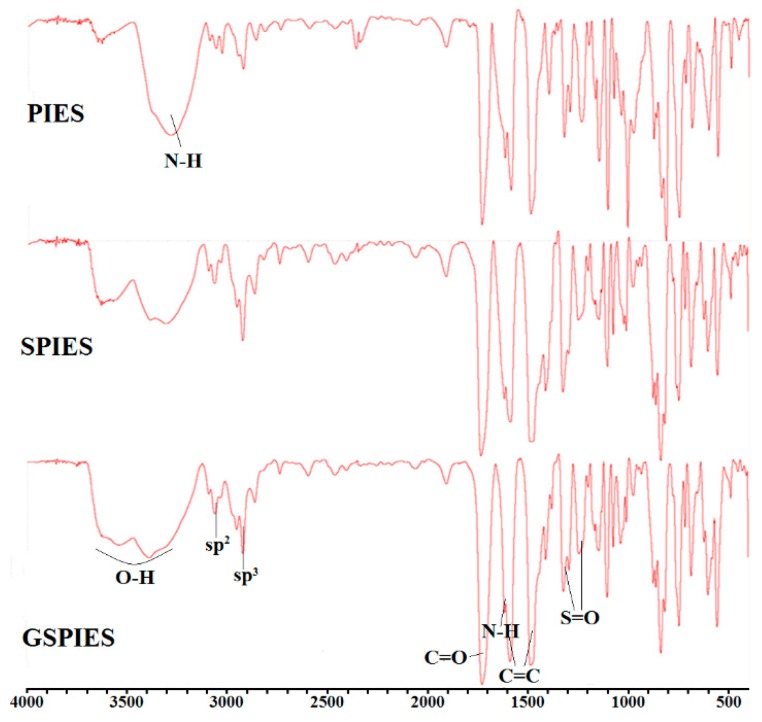
FT-IR of PIES, SPIES and GSPIES.

In the FT-IR study (Figure 4), the characteristic absorption at 1315 and 1120 cm^−1^ was assigned to the stretching vibration (S=O). The N–H stretching showed at 3300 cm^−1^ and overlapped at 3500 cm^−1^ by water in the sulfonated polymer. Moreover, N–H bending appeared at 1610 cm^−1^ and the spectra of aromatic C=C showed at 1470 and 1580 cm^−1^.

### 3.2. Properties of Membranes

**Figure 5:**
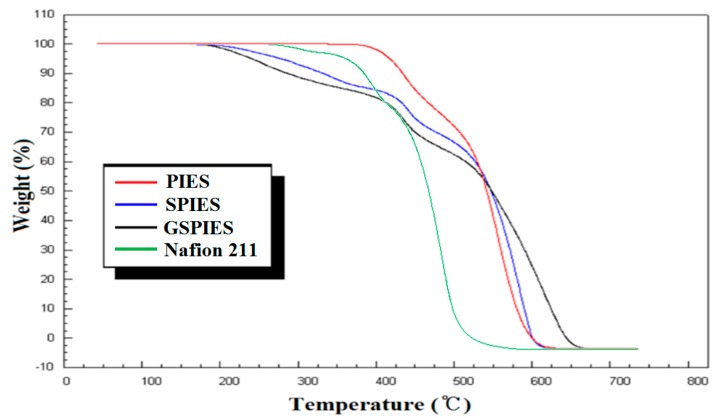
Thermooxidative stabilities of PIES, SPIES and GSPIES.

Thermooxidative stabilities of PIES, SPIES, GSPIES and Nafion211^®^ were tested using thermogravimetric analysis (TGA) under air atmosphere, and their results are shown in Figure 5.

PIES polymer was structured with isatin unit and showed relatively good thermal stability up to 400 °C. The first weight loss of SPIES and GSPIES occurred over 200 °C because of the decomposition of sulfonic acid groups. The second weight loss occurred around 400 °C because of the degradation of the polymer main chain. These polymers showed lower thermal stability than that of Nafion 211^®^. These results showed similar behaviors in other works [34,36]. Sulfonated polymers of SPIES and GSPIES were soluble in polar aprotic solvents such as DMAc, DMSO and NMP, *etc.*, and cast 25 μm film from DMSO solution to form light brown transparent films. IECs of the SPIES and GSPIES membranes were 1.46 and 0.71 meq/g respectively, and that of Nafion 211^®^ was a low value of 0.91 meq/g.

**Figure 6:**
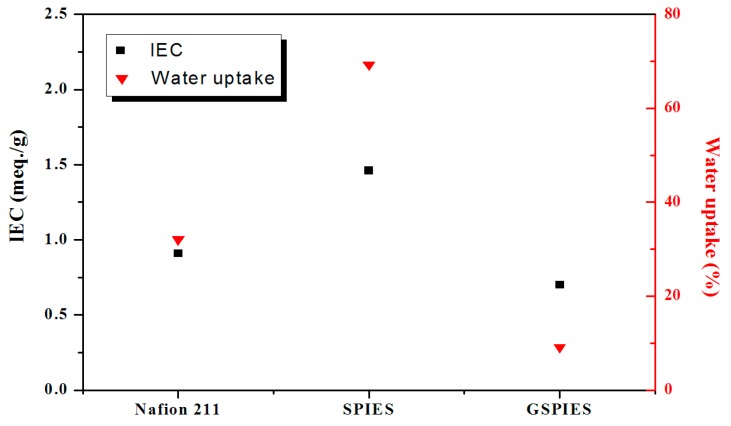
Ion exchange capacity (IEC) and water uptake of SPIES and GSPIES compared with Nafion 211^®^.

The water uptakes of the SPIES and GSPIES membranes were 69.3% and 9.12%, and that of Nafion 211^®^ was 32.1% at 80 °C under, 100% RH (Figure 6). The dimensional stability of membranes is an important factor when subjected to changes in temperature and humidity. The dimensional stability of the membrane was determined by through-plane (Δ*t*) and in-plane (Δ*l*) of the membranes under their RH100% and dry states. The dimensional changes of SPIES and GSPIES membranes resulted in Δ*t* values of 22.0% and 8.1%, and Δ*l* values of 10.2% and 5.0%, respectively (Table 1). 

**Figure 7:**
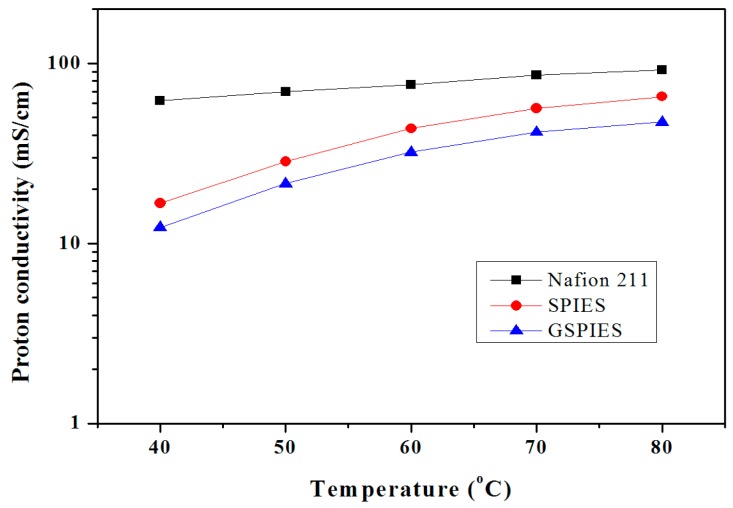
Proton conductivity of membranes at 40–80 °C under 80% RH.

A small change of dimensional stability is desirable behavior during operation of PEMFC. The proton conductivities of membranes were measured by a through-plane membrane test system with AC impedance spectroscopy. The conditions changed from 30 to 80 °C under 80% RH, and at 80 °C under 30%–90% RH while constant alternating current was applied to the anode and cathode at both electrodes (Figure 7).

**Figure 8:**
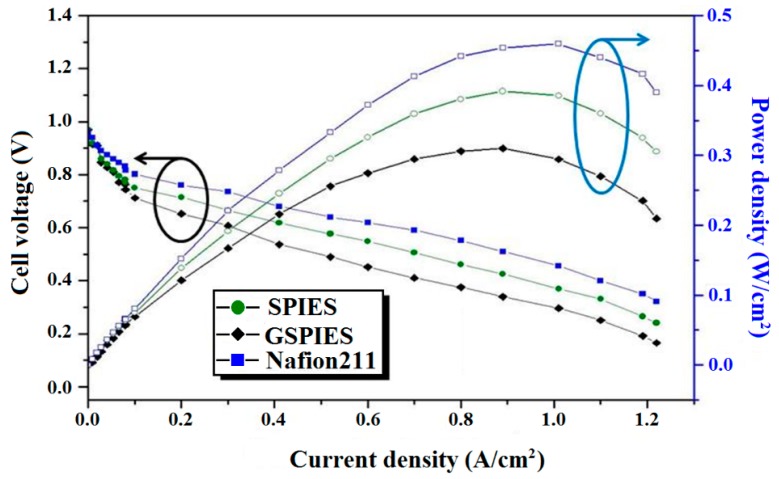
Cell performance of the membranes.

The proton conductivities of SPIES and GSPIES membranes were 65.4 and 47.2 mS/cm respectively at 80 °C with 80% RH, while Nafion 211^®^ is 104.5 mS/cm at same condition. Figure 8 showed the polarization and power density curves for SPIES and GSPIES under 100% RH conditions (RHa/RHc = 100%/100%).

The maximum power densities of SPIES, GSPIES and Nafion 211^®^ were about 0.39, 0.31 and 0.46 W·cm^−2^, respectively. The single cell performance of GSPIES membrane is relatively lower than that of SPIES because of low IEC value. The conductivities of membranes increased as temperature increased, and the membrane with higher IEC values had higher proton conductivity. The membranes of carbon-carbon spiro structure SPIES and GSPIES were shown the meaningful results, which have relatively high conductivity and low dimensional stability.

**Figure 9:**
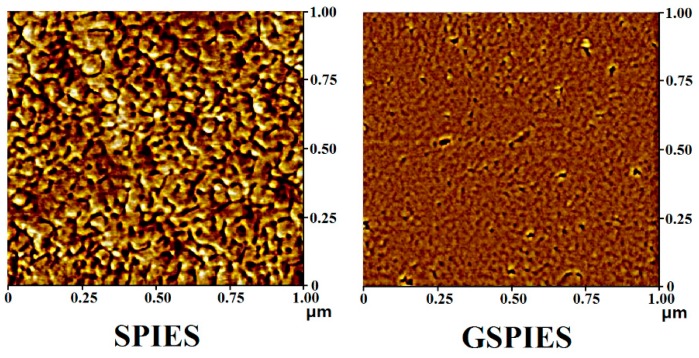
Atomic force microscopy images of SPIES and GSPIES.

The membrane morphology was observed by atomic force microscope (AFM) as shown in the phase images in Figure 9. The images presented that the surface patterns reflecting the hydrophilic-hydrophobic microphase separations were clearly discriminable bright and dark areas of membrane. The bright domains indicate the hydrophobic aromatic segments and the dark domains indicate hydrophilic sulfonic acid groups containing the absorbed water in air. The inter-connected hydrophilic conducting channels are helpful to the movement of protons. In dried membrane condition, the Young’s modulus of SPIES and GSPIES were 1169 and 1185 Mpa compared with 208 MPa of Nafion 211. These morphological results agree well with dimensional stability and the proton conductivity properties. All these properties indicate that SPIES and GSPIES are one of the most promising materials for using proton exchange membrane fuel cells.

## 4. Conclusions

A series of poly(isatin-ethersulfone) polymers were synthesized from isatin and bis-2,6-dimethylphenoxyphenylsulfone by superacid catalyzed polyhydroxyalkylation reactions. We designed and synthesized bis-2,6-dimethylphenoxyphenylsulfone, which is structured at the meta position steric hindrance by two methyl groups, because this structure minimized crosslinking reaction during super acid catalyzed polymerization. In addition, sulfonic acid groups were structured in both side chains and main chains to form better polymer chain morphology and improve proton conductivity. The sulfonation reactions were performed in two steps which are: in 3-bromo-1-propanesulfonic acid potassium salt and in con. sulfuric acid. These membranes of SPIES and GSPIES were 1.46 and 0.71 meq/g and that of Nafion 211^®^ was 0.91 meq/g. Membranes showed good thermal stability and low water uptake compared with Nafion 211^®^. The proton conductivities of SPIES and GSPIES are still lower than that of Nafion 211^®^ as shown in the typical hydrocarbon membranes. The chemical structures of monomer and polymer matrices are significantly dependent and well correlated in terms of their properties. The chemical structures of SPIES and GSPIES are desirable for fuel cell membrane application.

## Figures and Tables

**Table 1:** Properties of sulfonated and grafting polymer and Nafion211^®^.

Polymer	SPIES	GSPIES	Nafion 211^®^
Theoretical IEC (meq./g)	1.49	1.40	-
Titrated IEC (meq./g)	1.46	0.71	0.91
Water uptake ^a^ (%)	69.3	9.12	32.1
Δ*t* ^b^ (%)	22.0	8.1	14.1
Δ*l* ^b^ (%)	10.2	5.0	13.8
Young‘s modulus ^c^ (Mpa)	1,169	1,185	208
Proton conductivity ^d^ (mS/cm)	65.4	47.2	104.5

^a^ Water uptake at 30 °C; ^b^ Dimensional changes at 80 °C in water; ^c^ Dry condition; ^d^ Proton conductivity at 80 °C under 90% RH.
